# Second Language Proficiency Modulates the Dependency of Bilingual Language Control on Domain-General Cognitive Control

**DOI:** 10.3389/fpsyg.2022.810573

**Published:** 2022-02-10

**Authors:** Qiping Wang, Xinye Wu, Yannan Ji, Guoli Yan, Junjie Wu

**Affiliations:** ^1^Key Research Base of Humanities and Social Sciences of the Ministry of Education, Tianjin Normal University, Academy of Psychology and Behavior, Tianjin, China; ^2^Faculty of Psychology, Tianjin Normal University, Tianjin, China; ^3^Tianjin Social Science Laboratory of Students’ Mental Development and Learning, Tianjin, China

**Keywords:** bilingual language control, domain-general cognitive control, global control, local control, second language proficiency

## Abstract

The relationship between bilingual language control and domain-general cognitive control has been a hot topic in the research field of bilingualism. Previous studies mostly examined the correlation between performances of bilinguals in language control tasks and that in domain-general cognitive control tasks and came to the conclusions that they overlap, partially overlap, or are qualitatively different. These contradictory conclusions are possibly due to the neglect of the moderating effect of second language (L2) proficiency, that is, the relationship between bilingual language control and domain-general cognitive control might vary with the L2 proficiency of bilinguals. To examine this hypothesis, we recruited 36 unbalanced Chinese-English bilinguals to perform the Simon task (to assess domain-general cognitive control), Oxford Placement Test (to assess L2 proficiency), and picture naming tasks in single-and dual-language contexts (to evoke local and global language control). We find that Simon scores positively predicted switching costs in bilinguals with low L2 proficiency, but not in bilinguals with high L2 proficiency. Furthermore, Simon scores positively predicted mixing costs in bilinguals with high L2 proficiency, but not in bilinguals with low L2 proficiency. These results verify the moderating effect of L2 proficiency on the relationship between bilingual language control and domain-general cognitive control, that is, bilinguals with more proficient L2 rely on domain-general cognitive control less for local language control and more for global language control. This may imply a shift from local to global for the dependency of bilingual language control on domain-general cognitive control during the L2 development of bilinguals.

## Introduction

Language is the most essential and effective tool of communication and thinking for human beings. Learning and using two or more languages is increasingly common in modern society. Globally, more than half of the population is bilingual, across all age groups, all social classes, and most countries (Grosjean, 2013). Bilinguals seem effortless to juggle two languages in their minds. To achieve this, highly efficient mechanisms of bilingual language control (hereafter, referred to as language control) are needed for bilinguals according to the prevailing theories [Green, 1998; Adaptive Control Hypothesis or (ACH); Green and Abutalebi, 2013]. In the field of bilingualism, one of the important and unsettled issues is the relationship between language control and domain-general cognitive control (hereafter, referred to as cognitive control), which is the ability to regulate their behaviors by internal goals (Miller, 2000; Braver, 2012).

According to ICM (Green, 1998), bilinguals produce the target language by controlling the interference from the non-target language (specifically, inhibit it). When bilinguals intent to speak in their first language (L1) or second language (L2), lexical items from both languages are activated in parallel (Green, 1998; Meuter and Allport, 1999; Liu et al., 2020), interfering and competing with each other (Hermans et al., 1998; Green and Abutalebi, 2013). Each lexical item has an associated tag either for L1 or for L2. By virtue of the tags, bilinguals can globally exert inhibition over the lexical items of the entire language system that are unwanted in a certain situation. For a specific lexical item of the target language, bilinguals also need to exert inhibition over the interference from its translation equivalent at the local level. Furthermore, ICM claims an enhanced inhibition over the language of greater activation, which is usually the dominant L1 for unbalanced bilinguals.

Using the language switching paradigm, numerous studies provided evidence supporting ICM (e.g., Meuter and Allport, 1999; Ma et al., 2016; Declerck et al., 2017). In this paradigm, participants name pictures in the appropriate language according to a language cue (e.g., a red frame for L1, and a blue frame for L2). Unpredictable cues result in switch trials (where a different language is required to name a picture from the preceding trial, i.e., L1 → L2, L2 → L1) and non-switch trials (where the same language is required to name a picture from the preceding trial; i.e., L1 → L1, L2 → L2). Longer response times (RTs) and higher error rates (ERs) were found in switch trials than in non-switch trials (Meuter and Allport, 1999; Jylkkä et al., 2018, 2020; Timmer et al., 2018). The difference in performances of the switch and non-switch trials was termed *switching cost*. The switching cost of L1 was found to be larger than that of L2 in the literature (i.e., asymmetric switching costs), which has been taken as supporting evidence for ICM (e.g., Meuter and Allport, 1999; Ma et al., 2016; Gao et al., 2018). The rationale is that the dominant L1 was inhibited more strongly in the previous trials and required more time to overcome the inhibition in the current trial.

In the recent decade, an increasing number of studies focused on an essential research question: what is the relationship between language control and cognitive control (e.g., Linck et al., 2012; de Bruin et al., 2014; Calabria et al., 2015; Liu et al., 2016; Declerck et al., 2017; Jylkkä et al., 2018, 2020; Li et al., 2021). Some studies reported significant correlations (or covariation) of bilinguals’ performances in cognitive control tasks on the language switching costs and claimed overlapped mechanisms for language control and cognitive control (Linck et al., 2012; de Bruin et al., 2014; Liu et al., 2016; Declerck et al., 2017, 2021; Li et al., 2021). For instance, Linck et al. (2012) recruited 56 English-French-Spanish trilinguals to perform a language switching task and a Simon task. They found that bilingual speakers with smaller Simon scores that indicate the greater domain-general inhibitory control ability showed smaller language switching costs. Subsequently, Declerck et al. (2017) reported positive moderate to strong correlations between performances (indexed by switching costs) of bilinguals in language switching tasks and task-switching tasks across three experiments. These results support the view that bilinguals rely on cognitive control to achieve successful language selection and inhibition.

However, some studies failed to find the statistically significant correlation between bilinguals’ performances in cognitive control tasks and language switching costs (Calabria et al., 2012, 2015; Magezi et al., 2012; Prior and Gollan, 2013; Cattaneo et al., 2015; Prior et al., 2017; Segal et al., 2019). For example, Calabria et al. (2012) recruited 28 highly proficient Catalan-Spanish bilinguals to complete language switching tasks and non-verbal switching tasks. They observed the result of uncorrelated performances between the two tasks. Subsequently, they replicated the negative results (i.e., insignificant correlation) in a larger sample (*n* = 60) of highly proficient Catalan-Spanish bilinguals and further revealed the age-related effects in the non-verbal switching cost but not in the language switching cost (Calabria et al., 2015). These results lead to the conclusion that language control is qualitatively different from cognitive control.

In addition to the switching cost, researchers also examined the relationship between language control and cognitive control using more indices (Bobb and Wodniecka, 2013; Prior and Gollan, 2013; Cattaneo et al., 2015; Liu et al., 2019; Timmer et al., 2019). The mixing cost is thought to reflect global language control (which is also referred to as global control). To obtain this index, researchers added a picture-naming task of the single-language context in the experiment using the language switching task (i.e., the dual-language context). The extra time and errors were found in non-switch trials than trials in single-language contexts, which were termed as *mixing cost* (Christoffels et al., 2007; Gollan and Ferreira, 2009; Prior and Gollan, 2011; Fu et al., 2017; Jylkkä et al., 2018). Furthermore, the L1 mixing cost was always found larger than the L2 mixing cost in the literature, which is thought to reflect a larger global inhibition over the dominant language to guarantee successful production of the weaker language during the dual-language context (Christoffels et al., 2007; Gollan and Ferreira, 2009; Prior and Gollan, 2011; Fu et al., 2017; Jylkkä et al., 2018). The rationale is that the preceding trials and the current trials were named in the same language for non-switch trials and trials in single-language contexts, the difference of which is due to the language context. In the dual-language context, bilinguals have to exert more language control globally (i.e., global language control) over the trials (or the entire language system) compared to that in the single-language context, resulting in the mixing cost. In contrast, the switching cost is thought of as an index of local language control (which is also referred to as local control) in a way of trial-by-trial (Ma et al., 2016; Kleinman and Gollan, 2018; Wu et al., 2018), such as overcoming the local inhibition over the lexical item of non-target language in the preceding trials (Meuter and Allport, 1999; Linck et al., 2012; Declerck et al., 2019; Vorwerg et al., 2019; but see Bobb and Wodniecka, 2013, for alternative interpretations). Considering the existence of global and local language controls, some researchers have been focusing on the relationship between cognitive control and language controls at different levels (Prior and Gollan, 2013; Jylkkä et al., 2018, 2020; Segal et al., 2019).

For example, Prior and Gollan (2013) adopted the switching cost and the mixing cost as indices to evaluate bilinguals’ performances of language control and cognitive control. They recruited 104 bilinguals with high L2 proficiency (56 early Spanish-English bilinguals, 17 early Mandarin-English bilinguals, and 31 late but proficient Hebrew-English bilinguals) to perform the language and non-verbal switching tasks in a training and transfer paradigm. They found limited transfer facilitation from prior practice with the non-verbal switching task, which showed a reduction in mixing costs but not in switching costs. Furthermore, a significant correlation between performances of the language and non-verbal switching tasks was found only in the mixing cost but not in the switching cost. Jylkkä et al. (2018, 2020) adopted multiple tasks of cognitive control to predict performances of bilingual language control indexed by the switching cost and mixing cost. Due to the different aspects of cognitive control measured in tasks of cognitive control (e.g., Simon task, Flanker task, etc.), very mixed results were observed. However, it’s worth noting that, in their study of 2018, the statistical significance of positively predicting the performances of language control tasks by that of cognitive control tasks was only shown on the index of local language control (specifically L2 switching costs) rather than that of global language control (i.e., mixing costs). In contrast, opposite results were found in their study of 2020 that statistical significance of the same analysis was only shown on the index of global language control (specifically L1 mixing costs) rather than that of local language control (i.e., switching costs). After carefully examining the characteristics of the participants in these two studies, we found that the participants in the study of Jylkkä et al. (2018) seem less proficient in L2 than that in the study of Jylkkä et al. (2020). The averaged self-rated L2 proficiency were, respectively, 5.90 and 6.24 on a scale of 1–7 (1 for very unskilled and 7 for very skilled). Due to the lack of direct comparison between studies, no solid conclusion could be made from two groups of bilinguals with self-rated measures. However, these results at least suggested a possibility that the relationship between language control and cognitive control might differ in bilinguals with different L2 proficiency. This viewpoint might reconcile the inconsistent results of correlations between bilinguals’ performances of cognitive control and language control from a perspective of individual differences.

Indeed, it has been documented in the literature that L2 proficiency might modulate the mechanisms of language control (e.g., Costa et al., 2004, 2006; Filippi et al., 2014; Mouthon et al., 2019). For example, Costa et al. (2004) replicated the asymmetry of language switching costs in low-proficiency bilinguals (Experiment 1) but revealed symmetric switching costs in high-proficiency bilinguals (Experiments 2, 3, 4). Subsequently, Filippi et al. (2014) recruited native Italian speakers with various proficiency of English to perform the language switching task and found a smaller asymmetry of language switching costs in bilinguals with higher L2 proficiency. Furthermore, Mouthon et al. (2019) found the left middle frontal and left caudate areas (which are crucial regions for language control) were more involved during the language control task than cognitive control task for bilinguals with low L2 proficiency, whereas these brain regions were similarly involved in both tasks for the highly proficient group. These results suggest a need for considering L2 proficiency in examining the relationship between language control and cognitive control.

In the current study, we recruited native Chinese speakers with various proficiency of English to perform picture naming tasks in single-and dual-language contexts. The switching cost and mixing cost were used to index local control and global control, respectively. The Oxford Placement Test (OPT) was used to measure participants’ L2 proficiency (Allan, 2004; Lu et al., 2017). Additionally, the Simon task was adopted to measure their ability of cognitive control (specifically inhibitory control; Simon and Rudell, 1967; Bialystok et al., 2004; Wu et al., 2020). It’s worth noting that we chose the Simon task in the present study mainly for two reasons. First, the Simon task was one of the most frequently used tasks in the previous studies on the current issue (e.g., Linck et al., 2012; de Bruin et al., 2014; Liu et al., 2016; Prior et al., 2017; Jylkkä et al., 2018, 2020; Li et al., 2021). Second and more importantly, as aforementioned, research has addressed the involvement of inhibitory control over the non-target language during bilingual language production (see reviews by Green, 1998; Green and Abutalebi, 2013). Therefore, the Simon task chosen in the present study potentially has the theoretical value for uncovering the mechanisms of bilingual language control. We examined the hypothesis that the dependency of language control on cognitive control could be modulated by L2 proficiency. And if it does, we would further determine the exact L2 moderating effects related to local and global language controls. We expected divergence of predicting switching or mixing costs by Simon scores in bilinguals with various OPT scores.

## Materials and Methods

### Participants

As shown in Table 1, we recruited 36 Chinese-English unbalanced bilinguals (23 females) with a mean age of 24.53 years (*SD* = 5.20, range = 18–41). All participants were right-handed and had normal or corrected-to-normal vision. None of them reported any neurological disorders. The cognitive control ability of each participant was assessed by the Simon task. The average Simon score was 46 ms (*SD* = 30). Participants reported their L2 onset time (the age of starting to learn English as a second language) at the mean age of 8.80 years (*SD* = 2.47). The language switching experience was rated at the mean score of 2.69 (*SD* = 0.78) on a scale of 1–5 (1 for never, 5 for always). All participants rated their proficiency of L1 and L2 on a scale of 1–10 (1 for very unskilled, 10 for very skilled). Participants averagely scored 8.44 (*SD* = 1.03) and 6.24 (*SD* = 1.24) for Chinese and English proficiency, respectively. A paired *t*-test showed a significantly higher proficiency for Chinese than that for English (*t* = 8.66, *p* < 0.001), suggesting that unbalanced bilinguals were recruited in the present study. Only 28 participants took the College English Test Band 4 (CET-4; a standard English proficiency test for college students in China; full score = 710) and averagely scored 484 (*SD* = 48). We further assessed the English proficiency of each participant using the OPT with a full score of 100. The average OPT score was 71 (*SD* = 10).

**TABLE 1:** Mean values for individual difference measures.

Characteristics	Mean values (*n* = 36)
Age (years)	24.53 (5.20)
L2 onset time (years)	8.80 (2.47)
L1 self-rating scores	8.44 (1.03)
L2 self-rating scores	6.24 (1.24)
Language switching experience	2.69 (0.78)
CET 4 scores	484 (48)*
Simon scores (RT_*IC*–*C*_)	46 (30)
OPT scores (% accuracy)	71.28 (10.38)

L1, Chinese; L2, English. L1 self-rating scores, a scale of 1–10 (1 for very unskilled, 10 for very skilled). L2 self-rating scores, a scale of 1–10 (1 for very unskilled, 10 for very skilled). Language switching experience, a scale of 1–5 (1 for never, 5 for always). CET-4, College English Test-Band 4, a compulsory test measuring the English proficiency of undergraduate students in China with a full score of 710. *Only 28 participants took the CET-4. RT, response time (ms); IC, incongruent trials; C, congruent trials; OPT, Oxford Placement Test. The standard deviations (SDs) are shown in parentheses.

Additionally, due to the difficulty in visualizing the association between language control and cognitive control in bilinguals with various L2 proficiency levels when taking the OPT score as a continuous variable, we categorized participants into high-and low-proficiency groups based on the median of OPT scores for intuitive presentations. Two-sample *t*-tests were conducted to check if these two groups were matched on measures other than L2 proficiency. Only significant difference was showed in OPT scores (*t* = 7.17, *p* < 0.001) and marginally significant difference was showed in CET-4 scores (*t* = 1.91, *p* = 0.068). The averaged OPT scores for high-and low-proficiency groups were 79 (*SD* = 6) and 63 (SD = 6), respectively. The averaged CET-4 scores for high-and low-proficiency groups were 504 (*SD* = 57, *n* = 12) and 470 (*SD* = 33, *n* = 16), respectively. There was no significant difference between these two groups in the measures of age, L2 onset time, L1 and L2 self-ratings, and Simon scores (*t*s < 1).

All participants understood the experimental requirements before the experiment and signed the informed consent. This study has been approved by the Ethics Review Board of the Psychology Department of Tianjin Normal University and has strictly followed the relevant guidelines for human participants in the implementation process.

### Materials

In all, 34 standardized black and white line drawings were selected from Snodgrass and Vanderwart (1980), 4 of which were used as practice or fillers. Attributes of the chosen pictures are shown in Table 2, with English and Chinese norm data from Snodgrass and Vanderwart (1980) and Zhang and Yang (2003), respectively.

**TABLE 2:** Attributes for experimental materials.

	Frequency	Familiarity	Image agreement	Visual complexity
Chinese norms	46.47 (52.95)	4.57 (0.36)	3.49 (0.43)	2.33 (0.74)
English norms	59.67 (107.81)	3.65 (0.92)	3.45 (0.56)	2.74 (0.94)

*The standard deviations (SDs) are shown in parentheses. The frequency reported in the English and Chinese datasets (Snodgrass and Vanderwart, 1980; Zhang and Yang, 2003, respectively) is the frequency counts [or tokens, rather frequency per million words (fpm)] in each corresponding corpus (of 1.014 and 1.314 million words, respectively). The familiarity, visual complexity, and image agreement were scaled from 1 to 5 (1 for very unfamiliar/simple or low agreement, and 5 for very familiar/complex or high agreement).*

### Procedure

#### Picture-Naming Task

Before the experiment, the participants were instructed to familiarize themselves with the pictures and their corresponding Chinese and English names. Specifically, participants were instructed to browse through the pictures one by one for at least three runs. For the first two runs, each picture was presented with its Chinese or English name, respectively. Only one language (i.e., Chinese or English) was used during the entire run and the order of the language was counterbalanced across participants and kept consistent with that in the single-language context. After the second run, participants can choose to take the test directly or review the pictures and the corresponding names in both languages before the test. The test was conducted in the last run, which presented each picture without the name. Participants were required to name each picture in both Chinese and English. The correctness of the naming response was checked by the experimenter. The experimenter marked the pictures with incorrect responses and emphasized them to the participants before the test was re-taken. Only if all pictures were named correctly in both languages, the participant was allowed to enter the experiment.

In the formal experiment, the instructions were presented first. The participants were required to choose the correct language according to the color of the frame to name the picture quickly and accurately. The cue-language mappings were counterbalanced across participants. The process of each trial was as follows: firstly, the fixation point “+” of 300 ms was shown in the center of the screen, then the blank screen for 200 ms appeared, and then the pictures with a blue or red frame were presented. The participants named the pictures according to the language corresponding to the colors they were told in advance. When the participants reacted, the pictures disappeared, then either going on to the next trial or waiting for a blank screen of 1,000 ms. The longest time to present the picture on the screen was 2,200 ms. If participants did not make any response, three exclamation marks would appear in the center of the screen, and participants needed to press the space to enter the next trial. The experiment consisted of three parts: firstly, the single-language context of L1 and L2 required participants to name pictures only L1 or L2 (i.e., blocked naming or single-language trials); afterward, the dual-language context instructed participants to name pictures in L1 or L2 according to unpredictable cues and result in switch and non-switch trials; finally, the single-language context of L2 and L1 with reversed order was performed again. The order of the single-language context of L1 and L2 was also counterbalanced across participants. There were 120 trials in single-language contexts and 240 trials in the dual-language context, resulting in 360 trials in total.

#### Simon Task and Oxford Placement Test

Participants completed the Simon task to assess their cognitive control capability. They were instructed to ignore the location and only respond to the color of a square with the left or right key (e.g., the left key for the blue square, the right key for the red square). On neutral trials, the location of the square was in the middle of the screen. The location of the square was consistent with the required response key on congruent trials (e.g., a blue square appeared on the left side of the screen and the participants were required to press the left key), and opposite with the required response key on incongruent trials (e.g., a blue square appeared on right side of the screen and participants were required to press the left key). The difference between RTs on congruent and incongruent trials was calculated as the *Simon score*, which was interpreted as an indicator of the capability of cognitive control (Simon and Rudell, 1967; Bialystok et al., 2005; Paap and Greenberg, 2013; Wu et al., 2020).

In the OPT, there were 50 questions to test the English proficiency of participants. The scores were the percentage of correct answers, such that the higher the scores, the greater the English proficiency (Allan, 2004; Lu et al., 2017; Liu et al., 2021). In addition, a language history questionnaire (LHQ) was used to assess the linguistic background of participants (Schwieter and Sunderman, 2008; Declerck et al., 2021).

## Results

Before statistical analyses of RTs, we calculated the ER for each subject on each item and excluded trials that were named in a wrong language (2.97%), trials after naming in a wrong language (2.98%), trails in which the participant used the cued language but responded with the wrong word (2.13%), trials with hesitations (2.08%), trials of recording failures or absolute outliers (below 300 ms or above 2,200 ms, 3.48%), and trials of relative outliers (2.5 *SD*s above or below individual’s mean, 2.47%). Using the *lme4* (Bates et al., 2014) and *lmerTest* packages (Kuznetsova et al., 2017) in R (version 4.0.5),^1^ data of all participants were fitted and analyzed in linear mixed-effects models (LMEMs) for switching costs and mixing costs, respectively.

Additionally, to intuitively present the divergence of predicting switching or mixing costs by Simon scores in bilinguals with various L2 proficiency, we categorized participants into high-and low-proficiency groups based on the median of OPT scores and separately re-fitted and analyzed the LMEMs in each group. It is worth noting that statistical conclusions in the present study were made from all participants based on the regression models taking OPT as a continuous variable rather than from comparisons between two arbitrarily divided groups.

### Switching Cost (Local Control)

Log-transformed RTs were taken as the dependent variable. Language (L1, L2), Trial type (switch, non-switch), Simon score, OPT score, and their interactions were taken as the fixed effects. The ERs were taken as the control variable, including random intercepts for subjects and items (Barr et al., 2013). The significance (*p*-value) of fixed effects was determined based on Satterthwaite’s approximation (Kuznetsova et al., 2017). For this model, L1 and L2 were coded with contrast coding (L1 = –0.5, L2 = 0.5). Switch and non-switch trials were also coded with contrast coding (switch = 0.5, non-switch = –0.5). OPT and Simon scores were, respectively, centered at the sample mean.

The main effect of Language was significant (*t* = –17.06, *p* < 0.001), showing that naming in L2 was significantly faster than in L1 (989 and 1,083 ms, respectively). The main effect of Trial type was significant (*t* = 27.87, *p* < 0.001), which indicates that the RTs in switch trials were significantly longer than in non-switch trials (1,112 and 961 ms, respectively), yielding a switching cost of 151 ms. The interaction between Language and Trial type failed to reach significance (*t* = –1.57, *p* = 0.117), suggesting an apparent symmetry of switching costs (160 and 142 ms for L1 and L2, respectively). See Table 3 for more details.

**TABLE 3:** Estimated coefficients (logRT) from the mixed-effects model for examining the switching cost.

*Predictors*	*b*	*t*	*p*
(Intercept)	6.89	354.61	**<0.001**
Language	–0.09	–17.06	**<0.001**
Trial type	0.15	27.87	**<0.001**
ER	0.10	2.37	**0.018**
Language × Trial type	–0.02	–1.57	0.117
Simon	0.04	2.09	**0.037**
OPT	–0.01	–0.62	0.537
Language × Simon	0.01	1.61	0.108
Trial type × Simon	0.01	2.43	**0.015**
Language × OPT	0.00	0.54	0.586
Trial type × OPT	–0.00	–0.50	0.618
Simon × OPT	0.03	1.56	0.118
(Language × Trial type) × Simon	–0.01	–1.36	0.175
(Language × Trial type) × OPT	0.01	0.79	0.429
(Language × Simon) × OPT	–0.00	–0.73	0.466
(Trial type × Simon) × OPT	–0.01	–1.97	**0.049**
(Language × Trial type × Simon) × OPT	–0.02	–1.80	0.072

*logRT, log-transformed response time; b, raw (unstandardized) coefficient; bold values denote statistical significance at the p < 0.05 level; ER, error rate; OPT, Oxford Placement Test. OPT and Simon scores were, respectively, centered at the sample mean. Language was contrast-coded with L1 as –0.5 and L2 as 0.5. Trial type was contrast-coded with switch as 0.5 and non-switch as –0.5.*

**FIGURE 1:**
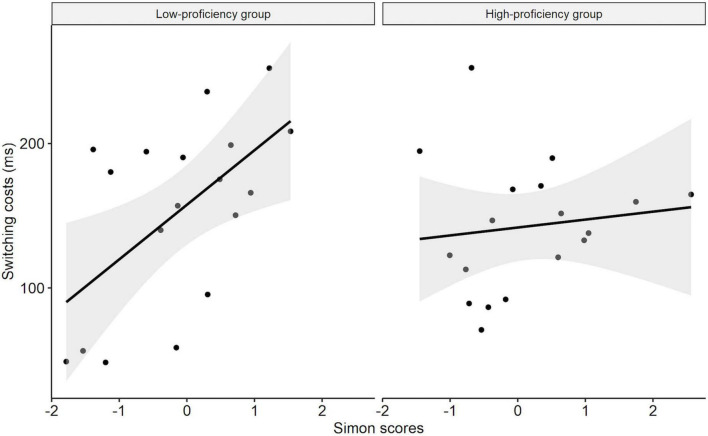
Relationship between inhibitory control and the switching cost modulated by OPT score.

There was a significant two-way interaction between Trial type and Simon score (*t* = 2.43, *p* = 0.015), showing that Simon scores predicted switching costs positively. In other words, as Simon scores increased, switching costs increased. Moreover, we found a significant three-way interaction between Trial type, Simon score, and OPT score (*t* = –1.97, *p* = 0.049), suggesting a negative moderating effect of OPT scores on the relationship between Simon scores and switching costs. In other words, the association between Simon scores and switching costs were weaker for the participants with higher OPT scores (Table 3). Additionally, we separately re-fitted the above mixed-effects model without OPT score for the high-and low-proficiency groups. The results showed a significant interaction between Simon score and Trial type in the low-proficiency group (*t* = 3.81, *p* < 0.001), whereas there was no significant interaction between Simon score and Trial type in the high-proficiency group (*t* < 1). This indicates that Simon scores positively predicted switching costs only in the low-proficiency group but not in the high-proficiency group (see Table 4 and Figure 1).

**TABLE 4:** Estimated coefficients (logRT) from the mixed-effects models for examining the switching cost, respectively, in low-and high-proficiency groups.

	Low-proficiency group			High-proficiency group		
*Predictors*	*b*	*t*	*p*	*b*	*t*	*P*
(Intercept)	6.93	261.02	**<0.001**	6.88	254.51	**<0.001**
Language	–0.09	–11.67	**<0.001**	–0.09	–12.29	**<0.001**
Trial type	0.15	19.59	**<0.001**	0.14	20.24	**<0.001**
Simon	0.03	0.99	0.320	0.06	2.13	**0.034**
ER	0.06	1.00	0.316	0.17	2.69	**0.007**
Language × Trial type	–0.03	–1.68	0.092	–0.01	–0.42	0.672
Language × Simon	0.02	2.11	**0.035**	0.00	0.29	0.771
Trial type × Simon	0.03	3.81	**<0.001**	–0.00	–0.16	0.870
(Language × Trial type) × Simon	–0.01	–0.35	0.723	–0.02	–1.39	0.165

*logRT, log-transformed response time; b, raw (unstandardized) coefficient; bold values denote statistical significance at the p < 0.05 level; ER, error rate. The Simon scores were centered at the sample mean. Language was contrast-coded with L1 as –0.5 and L2 as 0.5. Trial type was contrast-coded with switch as 0.5 and non-switch as –0.5. Participants were grouped into high-and low-proficiency groups based on the median score of the Oxford Placement Test (OPT).*

### Mixing Cost (Global Control)

Log-transformed RTs were taken as the dependent variable. Language (L1, L2), Trial type (non-switch, single-language), Simon score, OPT score, and their interactions were taken as the fixed effects. The ERs were taken as the control variable, including random intercepts for subjects and items (Barr et al., 2013). The significance (*p*-value) of fixed effects was determined based on Satterthwaite’s approximation (Kuznetsova et al., 2017). L1 and L2 were coded with contrast coding (L1 = –0.5, L2 = 0.5). Non-switch and single-language trials were also coded with contrast coding (non-switch = 0.5, single-language = –0.5). The OPT scores and Simon scores were, respectively, centered at the sample mean.

The main effect of Language was significant (*t* = –11.14, *p* < 0.001), showing that the RTs of L1 were significantly larger than that of L2 (949 and 892 ms, respectively). The main effect of Trial type was significant (*t* = 15.47, *p* < 0.001), indicating that the RTs in non-switch trials were significantly longer than that in single-language trials (961 and 880 ms, respectively), yielding a mixing cost of 81 ms. Additionally, the interaction between Language and Trial type was significant (*t* = –4.69, *p* < 0.001), suggesting that the L1 mixing cost (108 ms) was significantly larger than the L2 mixing cost (53 ms). See Table 5 for more details.

**TABLE 5:** Estimated coefficients (logRT) from the mixed-effects model for examining the mixing cost.

Predictors	*b*	*t*	*P*
(Intercept)	6.79	372.23	**<0.001**
Language	–0.06	–11.14	**<0.001**
Trial type	0.08	15.47	**<0.001**
ER	0.10	2.39	**0.017**
Language × Trial type	–0.05	–4.69	**<0.001**
Simon	0.03	1.68	0.093
OPT	–0.00	–0.26	0.797
Language × Simon	0.01	1.65	0.098
Trial type × Simon	0.01	1.92	0.055
Language × OPT	–0.00	–0.29	0.775
Trial type × OPT	–0.01	–2.10	**0.036**
Simon × OPT	0.03	1.38	0.166
(Language × Trial type) × Simon	0.01	1.21	0.225
(Language × Trial type) × OPT	–0.00	–0.05	0.959
(Language × Simon score) × OPT	0.02	3.13	**0.002**
(Trial type × Simon score) × OPT	0.03	4.23	**<0.001**
(Language × Trial type × Simon) × OPT	–0.02	–2.01	**0.044**

*logRT, log-transformed response time; b, raw (unstandardized) coefficient; bold values denote statistical significance at the p < 0.05 level; ER, error rate; OPT, Oxford Placement Test. OPT and Simon scores were, respectively, centered at the sample mean. Language was contrast-coded with L1 as –0.5 and L2 as 0.5. Trial type was contrast-coded with non-switch as 0.5 and single-language as –0.5.*

There was a marginally significant interaction between Trial type and Simon score (*t* = 1.92, *p* = 0.055), suggesting a trend that the higher Simon scores showed the greater mixing costs. In other words, as Simon scores increased, mixing costs tended to increase.

**FIGURE 2:**
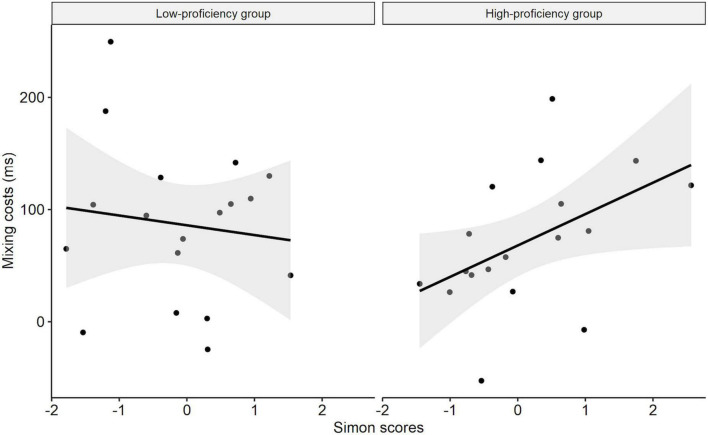
Relationship between inhibitory control and the mixing cost modulated by OPT score.

Moreover, we found a significant three-way interaction between Trial type, Simon score, and OPT score (*t* = 4.23, *p* < 0.001), indicating a positive moderating effect of OPT scores on the relationship between Simon scores and mixing costs. In other words, the association between Simon scores and mixing costs were stronger for the participants with higher OPT scores (Table 5). Additionally, we separately re-fitted the above mixed-effects model without OPT score for each group. As shown in Table 6, there was a significant interaction between Simon score and Trial type in the high-proficiency group (*t* = 3.55, *p* < 0.001), but not in the low-proficiency group (*t* = –1.09, *p* = 0.277). This indicates that Simon scores positively predicted mixing costs only in the high-proficiency group but not in the low-proficiency group (see Figure 2).

**TABLE 6:** Estimated coefficients (logRT) from the mixed-effects models for examining the mixing cost, respectively, in low-and high-proficiency groups.

	Low-proficiency group			High-proficiency group		
*Predictors*	*b*	*t*	*p*	*b*	*t*	*p*
(Intercept)	6.81	275.14	**<0.001**	6.74	274.20	**<0.001**
Language	–0.06	–7.91	**<0.001**	–0.05	–7.32	**<0.001**
Trial type	0.09	11.62	**<0.001**	0.07	10.14	**<0.001**
Simon	0.02	0.63	0.529	0.04	1.79	0.073
ER	0.06	0.99	0.320	0.16	2.59	**0.010**
Language × Trial type	–0.03	–2.29	**0.022**	–0.07	–4.71	**<0.001**
Language × Simon	0.01	1.43	0.153	0.00	0.57	0.565
Trial type × Simon	–0.01	–1.09	0.277	0.03	3.55	**<0.001**
(Language × Trial type) × Simon	0.02	1.10	0.272	0.01	1.00	0.317

*logRT, log-transformed response time; b, raw (unstandardized) regression coefficient; bold values denote statistical significance at the p < 0.05 level; ER, error rate; OPT, Oxford Placement Test. OPT and Simon scores were, respectively, centered at the sample mean. Language was contrast-coded with L1 as –0.5 and L2 as 0.5. Trial type was contrast-coded with non-switch as 0.5 and single-language as –0.5. Participants were grouped into high-and low-proficiency groups based on the median score of the Oxford Placement Test (OPT).*

## Discussion

In the present study, we aimed to examine whether L2 proficiency would modulate the relationship between language control and cognitive control. We recruited Chinese-English bilinguals with various English proficiency (assessed by OPT) and inhibitory control ability (assessed by Simon task) to perform the picture naming task in the dual-language and single-language contexts. The switching cost and mixing cost were adopted as indices for local and global language control, respectively. It was found that L2 proficiency indeed modulates the relationship between language control and cognitive control. To be specific, the switching cost was positively predicted by the Simon score in the low-proficiency group but not in the high-proficiency group, whereas the mixing cost was positively predicted by the Simon score in the high-proficiency group but not in the low-proficiency group. The results and implications are discussed as follows.

### Local Language Control and Domain-General Cognitive Control

In the low-proficiency group, we found that the switching cost was positively predicted by the Simon score. Previous studies also reported significant correlations between language switching costs and performances of bilinguals in tasks of cognitive control (Linck et al., 2012; Declerck et al., 2017; Jylkkä et al., 2018, 2020; Timmer et al., 2018; Li et al., 2021). For example, Linck et al. (2012) recruited participants to perform language switching tasks among their dominant English, the less proficient French, and the least proficient Spanish. They reported the first evidence that language switching costs could be predicted by bilinguals’ performances in the cognitive control task (specifically, Simon scores). Furthermore, subsequent studies found positive correlations between language and non-verbal switching costs in unbalanced bilinguals (Declerck and Grainger, 2017) and trilinguals (Timmer et al., 2018). These results were obtained from unbalanced bilinguals or trilinguals who were switching between dominant and less dominant languages. In the present study, we replicated the positive correlation between the language switching cost and the Simon score in low-proficiency Chinese-English bilinguals. According to the study of Filippi et al. (2014), unbalanced bilinguals who were less proficient in L2 showed larger switching costs in the dominant language during the language switching task, indicating modulating effect of L2 proficiency on the mechanisms of local language control. Thus, our results might imply recruitment of domain-general cognitive control for less proficient bilinguals in resolving trial-by-trial language conflicts at the local level of specific lexical items.

However, in the high-proficiency group, the switching cost could not be predicted by the Simon score. It is consistent with the studies that failed to find the correlation between language switching costs and performances of bilinguals in tasks of cognitive control (Calabria et al., 2012, 2015; Magezi et al., 2012; Prior and Gollan, 2013; Cattaneo et al., 2015; Branzi et al., 2016; Jylkkä et al., 2018, 2020; Segal et al., 2019; Timmer et al., 2019). For example, Timmer et al. (2019) examined the performances of highly proficient Catalan-Spanish bilinguals in the language and non-verbal switching tasks before and after a short-term training of language switching or single-language naming. They did not find significant correlations between language and non-verbal switching costs either at the pre-test or at the post-test. Similar results were found in other studies with highly proficient bilinguals (Calabria et al., 2012; Cattaneo et al., 2015, 2019; Branzi et al., 2016). These results resonate with the idea that, compared with the less proficient bilinguals, highly proficient bilinguals may develop different mechanisms of language control (Costa and Santesteban, 2004; Costa et al., 2006), which might not rely much on domain-general cognitive control to resolve language conflicts at the local level of specific lexical items.

Taken together, we found a negative moderating effect of L2 proficiency on the relationship between local language control and cognitive control. In other words, bilinguals with higher L2 proficiency showed less dependency of local language control on domain-general cognitive control, which is likely due to the different language control mechanisms in highly proficient bilinguals from that in less proficient bilinguals.

### Global Language Control and Domain-General Cognitive Control

We found the moderating effect of L2 proficiency in the relationship between the language mixing cost and the Simon score. In the high-proficiency group, the mixing cost was positively predicted by the Simon score. This result is in line with some previous studies (Prior and Gollan, 2013; Timmer et al., 2019; Jylkkä et al., 2020). For example, in highly proficient bilinguals, Prior and Gollan (2013) revealed a significant correlation between performances of language and non-verbal switching tasks only in mixing costs but not in switching costs. Similar correlational results were also reported in subsequent studies with highly proficient bilinguals (Timmer et al., 2019; Jylkkä et al., 2020). The results found in the present study confirmed the dependency of global language control on the domain-general cognitive control in Chinese-English bilinguals with relatively high proficiency in English.

However, in the low-proficiency group, we found that the mixing cost could not be predicted by the Simon score. It is in line with the previous studies that failed to find the correlation between the mixing cost and performances of bilinguals in non-verbal control tasks (Cattaneo et al., 2015; Jylkkä et al., 2018, 2020; Segal et al., 2019). These studies suggest little recruitment of cognitive control for bilinguals to exert global language control, which is supported by the maintenance of goal representation of the target language in working memory (Engle, 2002; Linck et al., 2020). The present study revealed negative results only in the low-proficiency group but not in the high-proficiency group, which may suggest that, compared with the bilinguals with higher L2 levels, the bilinguals with lower L2 levels are less proficient to recruit cognitive control to exert global language control over the interference from the entire system of the non-target language.

In short, we found that Simon scores positively predicted the mixing cost in the highly proficient group but not in the less proficient group, which is likely due to the demand for higher L2 proficiency or more experience of L2 usage for bilinguals to adopt cognitive control to globally prevent cross-language interference.

### A Shifting Hypothesis on the Dependency of Language Control on Cognitive Control

In the present study, we found that the relationship between language control and cognitive control was modulated by L2 proficiency. Specifically, Simon scores in bilinguals with higher L2 proficiency showed a weaker association with switching costs but a stronger association with mixing costs. These results might imply a shift from local to global for the dependency of language control on cognitive control during the L2 development of bilinguals.

It has been documented in the literature that the mechanisms of bilingual language control adapt to the improvement of L2 proficiency (Meuter and Allport, 1999; Costa et al., 2006; Filippi et al., 2014; Mouthon et al., 2019). For example, Meuter and Allport (1999) divided participants into two groups. One group was relatively balanced bilinguals and another group was L1-dominant bilinguals. They found that the L1-dominant group showed the predicted asymmetric language switching costs, but the more balanced group did not. Besides, Filippi et al. (2014) further reported that the asymmetry of language switching costs in bilinguals decreased with the improvement of L2 proficiency. These results might imply bilinguals rely less on local language control (indexed by switching costs) with the improvement of L2 proficiency. Furthermore, from a development perspective, previous studies have revealed the correlation between cognitive control and L2 development (Lu et al., 2017; Blom, 2019; Giguere et al., 2022). Blom (2019) found that the Flanker effects significantly predicted bilingual children’s L2 receptive vocabulary 1 year later, showing that better cognitive abilities lead to higher L2 vocabulary scores. Besides, Giguere et al. (2022) reported that L2 vocabulary scores were higher in bilingual children with better ability of cognitive control (i.e., smaller Flanker effect). These findings suggest that L2 proficiency may resolve some of the inconsistencies in the studies examining the relationship between bilingualism and cognitive control.

Some neuroscience studies also reported that compared with the less proficient bilinguals, the more proficient bilinguals had a decreased resting-state functional connectivity of the left anterior cingulate cortex and right middle frontal gyrus with other ROIs, and increased connectivity in the left insula, bilateral fusiform gyrus, left Para hippocampal region, and right putamen (Grant et al., 2015; Sun et al., 2019; Wang et al., 2020). Besides, it was found that bilinguals reconfigured their brain network to achieve language and non-verbal control, during which the reconfiguration efficiency was mediated by L2 proficiency (Wu et al., 2019). Critically, a recent longitudinal resting-state fMRI study revealed that with increased exposure to the L2, nodal betweenness in language control areas, such as the dorsal anterior cingulate cortex (dACC), decreased and connectivity between dACC and pre-supplementary motor area increased. These neural changes were correlated with participants’ behavioral performance on the language switching task, suggesting that the language control network in the resting brain could be modulated by long-term L2 learning (Liu et al., 2021).

In summary, the L2 development of bilinguals will lead to the change of their language structure and processing, which requires a dynamic and adaptive language control process (Abutalebi et al., 2013). This may imply a shift from local to global for the dependency of language control on cognitive control during the L2 development of bilinguals.

## Limitations

Although our results offer an insight into the controversial issue of the relationship between language control and cognitive control, there are some limitations in the present study that should be addressed in the future. For example, only the Simon task was adopted in the present study for measurement of inhibition, leaving it unknown how the other aspects of cognitive control (e.g., updating and shifting in the theoretical framework proposed by Miyake et al., 2000) contribute to the language control in bilinguals with various L2 proficiency. Furthermore, some recent studies failed to find good convergent validity of the non-verbal interference tasks (e.g., Simon and Flanker tasks) and claimed that the inhibition measured by these tasks might be task-specific rather than domain-general (Paap and Sawi, 2014; Rey-Mermet et al., 2018; Paap et al., 2020). Some researchers further questioned inhibition as a psychometric construct (Rey-Mermet et al., 2018). It is worth noting that the inhibition is collapsed into the common executive function in the latest framework of Friedman et al. (2008), Miyake and Friedman (2012), and Friedman and Miyake (2017). Future studies may adopt multiple tasks to extract latent variables for the whole profile of cognitive control and obtain a more comprehensive understanding of the current issue.

## Conclusion

The current study was designed to test the hypothesis that L2 proficiency modulates the relationship between cognitive control and language control. We found that Simon scores positively predicted the switching cost in bilinguals with low L2 proficiency but not in bilinguals with high L2 proficiency. Besides, Simon scores showed a positive prediction on the mixing cost in the high-proficiency group but not in the low-proficiency group. These results verify the moderating effect of L2 proficiency on the relationship between cognitive control and language control, demonstrating that bilinguals with more proficient L2 rely on cognitive control less for local language control and more for global language control. This may reveal a shift from local to global for the dependency of language control on cognitive control during the L2 development of bilinguals. The current study provides a new perspective to illuminate the controversial relationship between language control and cognitive control.

## Data Availability Statement

The raw data supporting the conclusions of this article will be made available by the authors, without undue reservation.

## Ethics Statement

The studies involving human participants were reviewed and approved by the Ethics Review Board of the Psychology Department of Tianjin Normal University. The patients/participants provided their written informed consent to participate in this study.

## Author Contributions

QW: conceptualization, data curation, formal analysis, investigation, methodology, software, visualization, writing—original draft, and writing—review and editing. XW: data curation, investigation, and visualization. YJ: data curation and visualization. GY: conceptualization. JW: conceptualization, funding acquisition, supervision, and writing—review and editing. All authors contributed to the article and approved the submitted version.

## Conflict of Interest

The authors declare that the research was conducted in the absence of any commercial or financial relationships that could be construed as a potential conflict of interest.

## Publisher’s Note

All claims expressed in this article are solely those of the authors and do not necessarily represent those of their affiliated organizations, or those of the publisher, the editors and the reviewers. Any product that may be evaluated in this article, or claim that may be made by its manufacturer, is not guaranteed or endorsed by the publisher.

## Footnotes

1
https://www.r-project.org
